# Neuromuscular fatigue and muscle damage following a simulated singles badminton match

**DOI:** 10.1007/s00421-023-05148-w

**Published:** 2023-02-10

**Authors:** Zengyuan Lin, Anthony J. Blazevich, Chris R. Abbiss, Jodie Cochrane Wilkie, Kazunori Nosaka

**Affiliations:** 1 Centre for Human Performance, School of Medical and Health Sciences, Edith Cowan University, Joondalup, WA Australia; 2 The Badminton Association of Western Australia, 130 Kingsway, Madeley, WA 6065 Australia

**Keywords:** Maximal voluntary contraction, Electrical stimulation, Voluntary activation, Muscle soreness, Lunge

## Abstract

**Purpose:**

To understand muscle damage in badminton, changes in neuromuscular function were investigated after simulated badminton singles matches performed by ten state-level male players.

**Methods:**

Each participant played eight matches and measurements were taken before, immediately after, and 1 and 24 h after each match. Maximal voluntary isometric contraction (MVC) torque of the knee extensors and flexors, voluntary activation (VA) during MVC and torques generated by doublet (*T*_Doublet_), 20 (*T*_20_) and 80 Hz (*T*_80_) electrical stimulations of the knee extensors were measured from the dominant leg (the racket-hold arm side). Muscle soreness was assessed by a 100-mm visual analogue scale from both legs. The number of lunges performed by each participant in each match was analysed by videos, and its relations to other measures were examined.

**Results:**

Pre-match knee extensor and flexor MVC torques were 278.4 ± 50.8 Nm and 143.0 ± 36.2 Nm, respectively. Knee extensor MVC torque of the dominant leg decreased immediately (12.0 ± 2.9%) and 1 h post-match (16.0 ± 3.2%), but returned to baseline at 24 h post-match. VA (11.4 ± 2.9%), *T*_Doublet_ (13.1 ± 6.0%), *T*_20_ (31.1 ± 12.3%) and *T*_80_ (25.5 ± 7.9%) decreased (*p* < 0.01) immediately post-match but recovered by 24 h post-match. A significant correlation (*r* = − 0.64, *p* < 0.01) was observed between the total number of lunges performed in a match (160–240 times) and the magnitude of decrease in MVC torque (6.4–14.7%). Muscle soreness developed more (*p* < 0.05) for the dominant (51.5 ± 11.6 mm) than the non-dominant leg (18.8 ± 8.6 mm).

**Conclusion:**

Muscle damage induced by singles badminton matches was minimal, but the more the lunges are performed, the greater the neuromuscular fatigue.

## Introduction

Badminton is the world’s fastest racket sport with the shuttlecock reaching a maximum velocity of 100 m·s^−1^ (360 km·h^−1^) with an average velocity being 50–75 m·s^−1^ during a match play (Cabello and Gonzalez-Badillo 2003; Chen and Chen 2008). In a badminton match, short duration movements (e.g. 2–9 s) interspersed with short rest periods (e.g. 5–15 s) are performed (Cabello and Gonzalez-Badillo 2003; Chen and Chen 2008; Tu 2007). It was reported that winning a point during intense rallies in singles badminton matches was strongly associated with the ability to move rapidly around the court (Lees 2003; Nadzalan et al. 2018a, b), with the lunges being one of the most frequently performed movements, accounting for 15% of all movements performed (Kuntze et al. 2010; Nadzalan et al. 2018a, b; Phomsoupha and Laffaye 2015).

Lunges require the activation of the quadriceps, hamstrings and gluteal muscles whilst the muscle–tendon units lengthen during an eccentric phase to produce the braking reaction force (Cronin et al. 2003; Jönhagen et al. 2009), which are normally executed by the leg on the same side as the arm holding the racket (Lees 2003). Thus, neuromuscular fatigue should be different between legs. It is also assumed that lunges are associated with muscle damage that is characterised by prolonged loss of muscle function and delayed onset muscle soreness (DOMS). With the high frequency of lunges executed during a badminton match (Kuntze et al. 2010; Nadzalan et al. 2018a, b; Phomsoupha and Laffaye 2015), it may be that the more the lunges are performed, the greater the loss of muscle function and DOMS after the match.

However, no previous studies have investigated the effects of the performance of lunge movements during matches on neuromuscular fatigue and muscle damage after a badminton match. Such information is useful in the development of specific (i.e. task targeted) fatigue and muscle damage minimization training programmes, and suggests a potential strategy by which a player might try to induce additional fatigue in an opponent.

Therefore, the aim of this study was to quantify changes in knee extensor and flexor neuromuscular function after a simulated 1-h badminton singles match in relation to the number of lunges performed in the match in state-level (Australian) badminton players. It was hypothesised that the muscle strength of the lower limb extensor muscles that are used to perform the lunges would decrease after the simulated match, and the magnitudes of changes in muscle strength and neuromuscular parameters would be associated with the number of lunges performed in the match, which is generally considered to indicate muscle damage (Girard and Millet 2009).

## Methods

### Participants

Ten competitive and highly competent male singles badminton players from the Western Australia badminton team, with at least 5 years of state-level playing experience, volunteered to participate in this study. They had just completed the season when they participated in the study; thus, the participants had been playing competitive tournaments before participation in the study. Their average (range) age, height and body mass were 26.4 ± 5.3 (19–33) years, 174.4 ± 8.6 (165–191) cm and 69.7 ± 8.5 (59.5–83) kg, respectively. The participants’ weekly training hours were 7.1 ± 1.2 h. The participants were requested to abstain from consuming caffeine for at least 6 h and alcohol at least 24 h prior to testing. Before participating in the study, participants were informed of the risks and procedures of the study and were provided informed consent. Ethics approval was obtained from the Edith Cowan University Human Research Ethics Committee.

### Study design and procedures

**Fig. 1:**
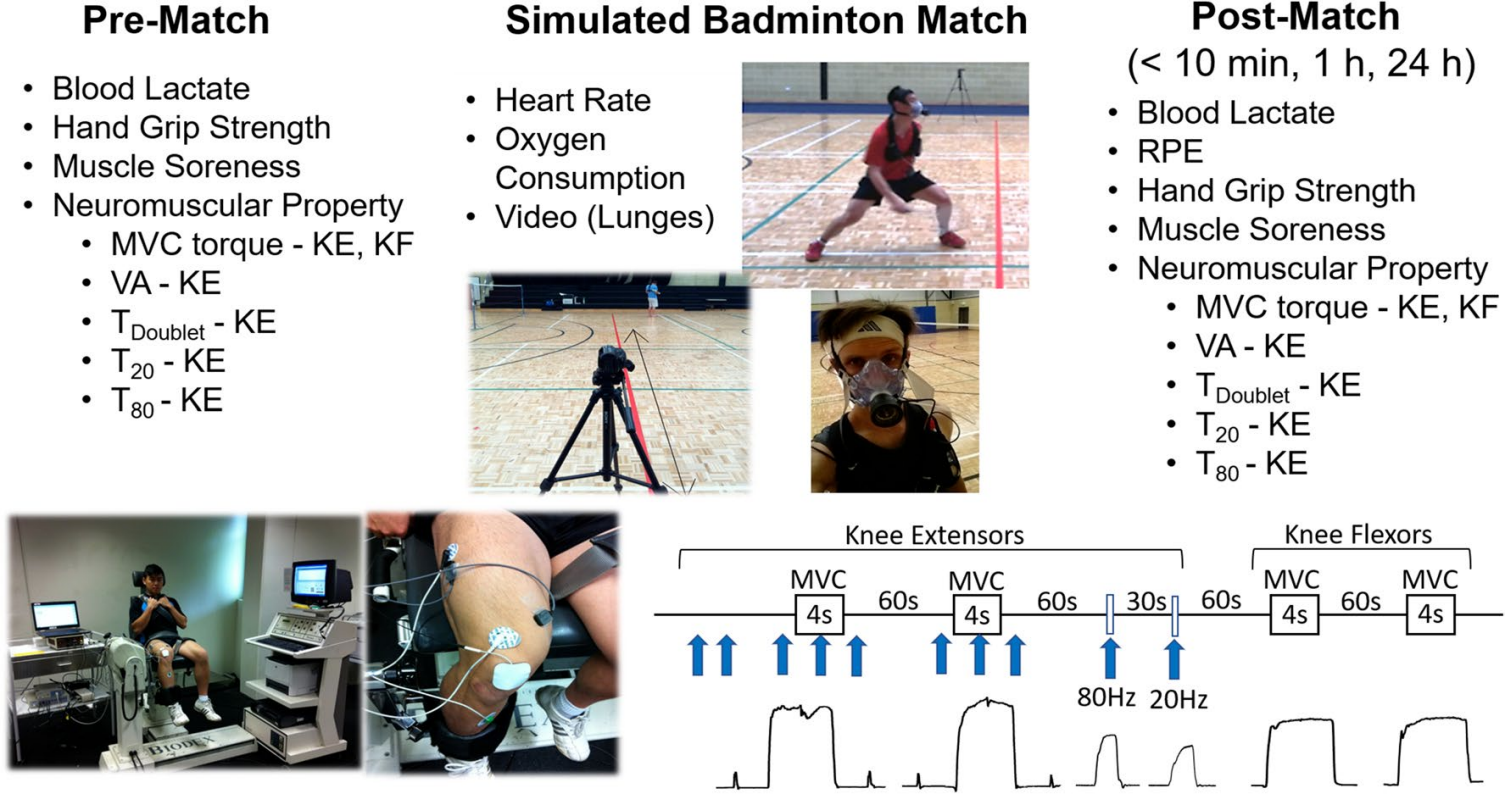
Experimental protocol. The measurements consisted of blood lactate, hand grip strength, muscle soreness, ratting of perceived exertion (immediately post-match only) and neuromuscular property such as maximal voluntary isometric contraction (MVC) torque of the knee extensors (KE) and flexors (KF), voluntary activation (VA) of the knee extensor during MVC, and torque generated by doublet (*T*_Doublet_), torque induced by 20 Hz (*T*_20_) and 80 Hz (*T*_80_) electrical stimulations of the knee extensors. These were measured prior to match (pre-match), within 10 min post-match, 1 h and 24 h post simulated badminton matches. During the simulated badminton matches, heart rate and oxygen consumption were measured, and the matches were recorded by videos to analyse lunges. The procedures for the neuromuscular property measurements are illustrated on the bottom of the figure

This study consisted of a total of ten sessions over 10 weeks for each participant, starting with a familiarisation session for all procedures, an incremental exercise test session and eight sessions of simulated badminton match play as detailed in the section below (simulated match). The outcome measures consisted of maximal voluntary isometric contraction (MVC) torque of the knee extensors and flexors, voluntary activation (VA) during MVC, and torques generated by doublet (*T*_Doublet_), 20 Hz (*T*_20_) and 80 Hz (*T*_80_) electrical stimulations of the knee extensors, muscle soreness assessed by a 100-mm visual analogue scale (VAS), handgrip strength, the number of lunges analysed by videos, and oxygen consumption in a match play (Fig. 1). The neuromuscular measurements were taken before, immediately after (within 10 min post-match), and 1 and 24 h after each match from the dominant leg (the racket-hold arm side). The non-dominant leg was only assessed for the MVC torque for the knee extensors and flexors following the measurement from the dominant leg. Muscle soreness was assessed from both legs prior to the neuromuscular measurements.

### Maximal aerobic capacity test (***V***O_2max_ test)

Participants performed a maximal aerobic capacity test (*V*O_2max_ test) on a treadmill in a standard room (temperature: 25 °C, relative humidity: 60%). During the test, participants began running at 10 km h^−1^ for 5 min at 0% gradient and the velocity was increased by 2 km h^−1^ every minute until 16 km h^−1^ maximum. Thereafter, the gradient of the treadmill was increased by 1% every minute and continued until volitional exhaustion was reached. Expired gas was measured breath-by-breath throughout the test with the use of a portable metabolic gas analyser (MetaMax^®^ 3B, Germany) and averaged every 30 s to determine *V*O_2max_ (Faude et al. 2007). Heart rate was continuously recorded and averaged over 5-s intervals using a Polar heart rate monitor (S610, Polar, Finland), and the data were transferred to a computer for further analysis.

### Simulated match

The ten participants were split into two groups (*n* = 5 for each group) based on their national ranking, with the first top five ranked participants in the first group and the remaining participants in the second group. The participants only played against the members of their own group. Within a group, participants played against each other twice; once as the assessed competitor and once as the non-assessed opponent due to the test equipment limitation. A total of eight 1-h simulated badminton matches were played by each participant (four sessions as assessed competitor and four sessions as non-assessed opponent) over a 4-week study period (i.e. two matches per week). A battery of tests (described below) was completed both before and after each match for the assessed competitor.

The simulated matches were standardised competitive badminton singles matches played under International Badminton World Federation rules, but were played for 1 h regardless of the scores. Participants were permitted to rest for a maximum of 5–10 s between points, and a maximum of 120 s between sets, timed by the investigator. All simulated matches were performed on indoor courts with an average air temperature of 24 ± 3 °C and were each scheduled to be played at the same time of the day.

For every match, oxygen consumption and muscle function measurements were performed on one participant, i.e. the assessed competitor, in a pair. In the subsequent match of the pair, testing was performed on the other participant (i.e. the non-assessed opponent became the competitor). Thus, these data were obtained from 4 matches per participant, and a total of 40 simulated badminton matches were analysed for the dependent variables. Heart rate (HR) was recorded during the match from both players, and blood lactate concentrations and rating of perceived exertion (RPE) were obtained before and immediately post-match from both players. All matches were video recorded for subsequent motion analysis as explained below.

### Heart rate (HR)

HR was recorded continuously during each badminton match using a heart rate monitor (S610, Polar, Finland) and was configured to connect wirelessly to a portable gas analyser (MetaMax® 3B, Cortex, Germany). Both the average HR during the entire 1-h match and the average HR during play by excluding the resting time were obtained and used for further analyses.

### Rating of perceived exertion (RPE)

The rating of perceived exertion was quantified by a Category Ratio 10 scale consisting of numbers between 0 (nothing at all) and 10 (maximal) (Neely et al. 1992). The participants were asked, “What was your overall perceived exertion?” at immediately post-match (Faude et al. 2007).

### Oxygen consumption

A portable metabolic gas analyser (MetaMax^®^ 3B, Cortex, Germany) was used to measure oxygen consumption during a match (Fig. 1). The device was strapped across the participant’s chest and the participant breathed through a Hans-Rudolph face mask that was connected to the analyser. Raw data were stored on the data logger of the metabolic system and transferred to a computer after each match for further analysis. *V*O_2_ was collected breath-by-breath and averaged over 5 s for each 1-h singles badminton match (Faude et al. 2007). This procedure was previously carried out by Faude et al. (2007) in a similar setup.

### Blood lactate

Blood lactate concentration was measured from a finger prick blood sample (Unistik 2 Normal: Owen Mumford, Oxford, UK) obtained from the non-racket holding hand prior to exercise and immediately after exercise, using a portable lactate analyser (Lactate Pro, Australia).

### Muscle function measurements

#### Hand grip strength

Hand grip strength for both arms (the dominant arm followed by the non-dominant arm) was measured with a manual handheld dynamometer (Lafayette Hand Dynamometer, USA) before and immediately, 1 h and 24 h after each simulated match. Two attempts were made for each hand with a rest interval of 1 min. The distance from the handle to the base of the dynamometer was kept consistent among the measures. The higher value of the two measurements for each arm was used for further analysis.

#### MVC torque and neuromuscular parameters

An isokinetic dynamometer (Biodex System 3 Pro, NY) was used to measure maximal voluntary knee extensor contraction (MVC) torque, with the trunk–thigh angle at 85° and the knee joint angle at 60° of flexion (0° corresponding to full knee extension). The measurement protocol began with warm-up consisting of submaximal contractions at self-perceived 30%, 60% and 80% of maximal isometric knee extensors and flexor contractions of the dominant leg first followed by the non-dominant leg. For the MVC measurements, participants were instructed to generate force ‘as fast and hard as possible’ twice over 4 s with a 60-s rest between contractions (Fig. 1). From the torque data, peak torque was calculated from each contraction and the higher value of the two was then used for further analyses.

During MVCs, maximal voluntary activation was estimated using the interpolated-twitch technique, with a doublet stimulation (10-ms inter-pulse interval, 200 µs pulse width, square wave) delivered by constant-current stimulator (DS7, Digitimer Ltd., Welwyn Garden City, UK) to the femoral nerve when the voluntary torque reached a plateau. A ‘control doublet’ was then delivered 5 s after the completion of each MVC for subsequent calculation of voluntary activation, using the formula (Girard et al. 2010):

$$\mathrm{Voluntary\, activation}= [1 - (\mathrm{superimposed\, doublet}/\mathrm{potentiated \,doublet})] \times 100$$

Paired-pulse electrical stimulations were delivered at progressively increasing intensity (10 mA increment) until a plateau of the evoked doublet twitch torque amplitude was observed. The intensity was then further increased by 20% and used throughout the testing session. Peak doublet torque (*T*_Doublet_) was assessed following a doublet stimulation during MVC execution (Girard et al. 2010). The 20:80 Hz stimulation ratio was then determined as the ratio of torques produced using high-frequency (80-Hz; *T*_80_) and low-frequency (20-Hz; *T*_20_) stimulations for 0.75 s (Girard et al. 2006). To determine the stimulation intensity, the current required to evoke 50% of the MVC using 20 Hz stimulation was determined and this intensity was used for all subsequent stimulations (Trajano et al. 2013).

**Fig. 2:**
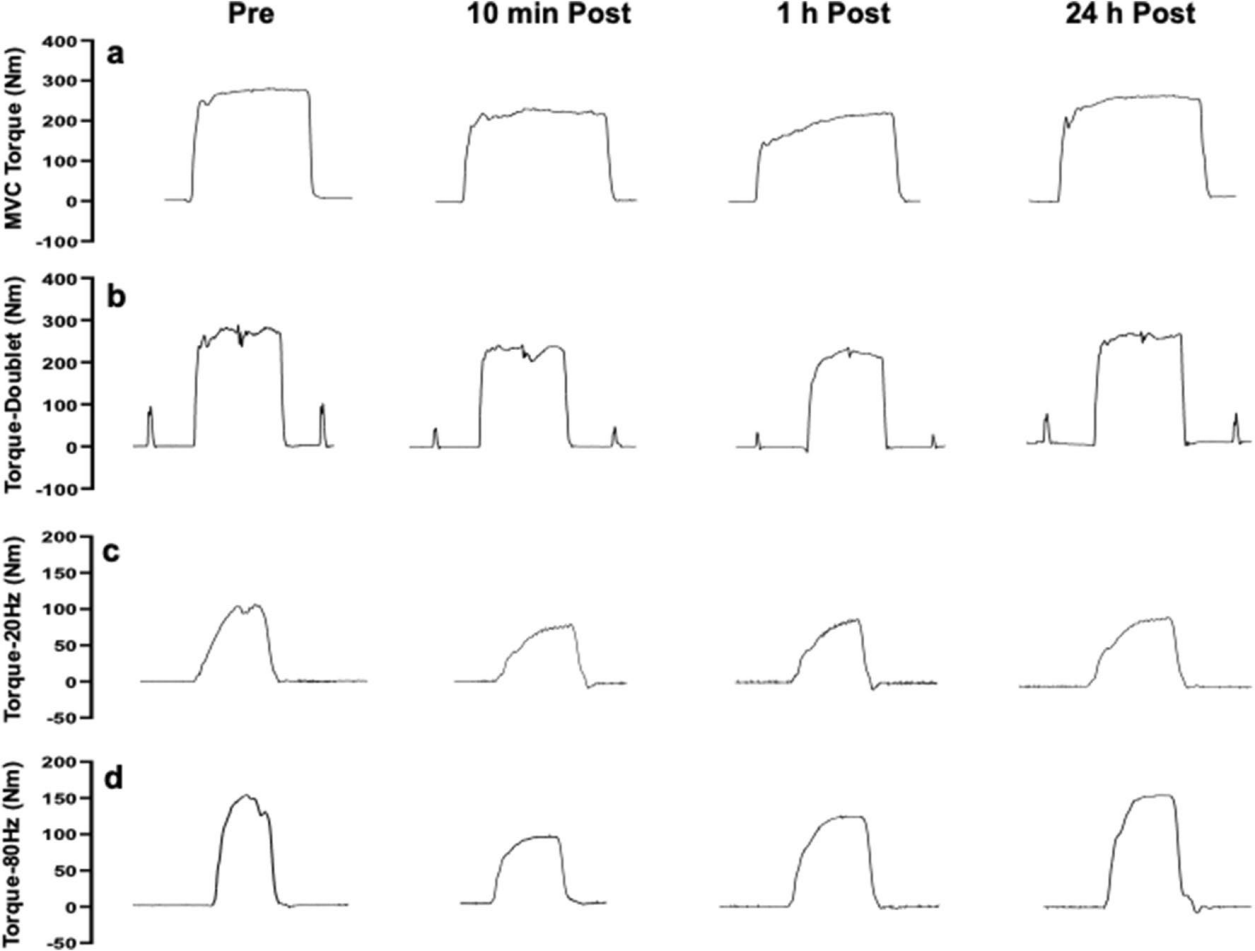
Typical force traces of MVC torque (**a**), and torque induced by doublet (**b**), torque induced by 20 Hz (**c**) and 80 Hz (**d**) electrical stimulation for the knee extensors of the dominant leg of a participant prior to a match (Pre), within 10 min after the match (10 min Post), and 1 h (1 h Post) and 24 h (24 h Post) after the match

Subsequently, knee flexor MVC torque was measured at a knee joint angle of 60° of flexion (0° corresponding to full knee extension), using the same procedures as for knee extensor testing without the electrical stimulations. The muscle function measurements from the dominant leg were taken pre-match, within 10 min (this is referred to as “immediately post-match” in the following sections), 1 h and 24 h post-match. For the measurements from the non-dominant leg, MVC torque of the knee extensors and knee flexors without electrical stimulation were performed at pre-match, 1 h and 24 h post-match. Typical force traces collected from the dominant leg of a participant over time are shown in Fig. 2.

### Muscle soreness

The level of muscle soreness was subjectively quantified using a 100-mm visual analogue scale (VAS) in which 0 indicated “no pain” and 100 represented “the worst pain imaginable” from both legs. The level of perceived pain of the quadriceps femoris, hamstring and gluteal muscles was assessed by a single leg forward lunge from each leg. The participants were specifically asked about soreness in each individual muscle to ensure that the value represented the soreness of each muscle rather than on other parts, or the whole, of the lower extremities (Gomes et al. 2014; Moreno-Perez et al. 2020).

### Video analyses

All matches were recorded with two video cameras (Sony HD 1080i, Japan) placed at two positions in each half of the court. The video was analysed to quantify the number of lunges performed by the participants by two separate investigators using Sports Code Pro software (Sportstec, USA). The mean of the two investigators was taken for further analyses. Lunges were classified as either a half lunge (the forward movement of the knee that did not exceed the position of the toe) or full lunge (the forward movement of the knee that went beyond the toe) as outlined by previous studies (Hong et al. 2014; Kuntze et al. 2010).

### Statistical analyses

A Shapiro–Wilk test was used to check the normality of the data prior to any analysis. A one-way analysis of variance (ANOVA) with repeated measures was used to assess changes in the variables measured before and after the matches (muscle soreness, MVC torque and other neuromuscular parameters). A two-way analysis variance (ANOVA) with repeated measures was used to compare the magnitude of changes of the MVC torque and neuromuscular parameters over time between dominant and non-dominant legs (using absolute values), and between knee extensors and flexors (using normalised values). Where significant interaction effects were detected, Bonferroni post hoc tests were performed. Pearson’s product-moment correlations were used to examine relationships between the number of lunges (total, full) and changes in MVC torque (Cronin et al. 2003). All statistical analyses were performed using SPSS version 25.0 (SPSS Inc., Chicago, IL, USA) with a significance level accepted at *p* ≤ 0.05, and the results are presented as mean ± standard deviation (SD).

## Results

### Participant physiological characteristics

Average *V*O_2peak_ and HR_max_ at *V*O_2peak_ during the *V*O_2max_ test were 57.0 ± 7.3 (range = 50–70) ml kg^−1^ min^−1^ and 187.6 ± 8.7 (173–195) bpm, respectively. Average *V*O_2_, HR and RPE during the simulated badminton matches were 44.3 ± 8.6 (37–56) ml·kg^−1^·min^−1^, 162.0 ± 10.6 (140–176) bpm and 7.0 ± 2.0 (5–9), respectively. The *V*O_2_ during the simulated badminton match represented approximately 80% of *V*O_2peak_, and HR represented approximately 84% of HR_max_. The blood lactate concentration was 1.8 ± 0.3 mM l^−1^ at pre-match and increased to 7.2 ± 1.3 (5.9–9.3) mM l^−1^ immediately post-match. Post-match CR-10 was 7.9 ± 0.9 (6.6–9.2).

### Muscle function

#### Handgrip strength

Handgrip strength of the dominant and non-dominant hands was 56.7 ± 12.9 (37.4–73.2) kg and 49.7 ± 9.6 (35.4–64.9) kg at baseline, and 56.6 ± 11.4 (42.3–73.1) kg and 50.3 ± 8.8 (39.5–67.1) kg immediately post-match, respectively. No significant changes in hand grip strength were detected after the match.

#### MVC torque

**Fig. 3:**
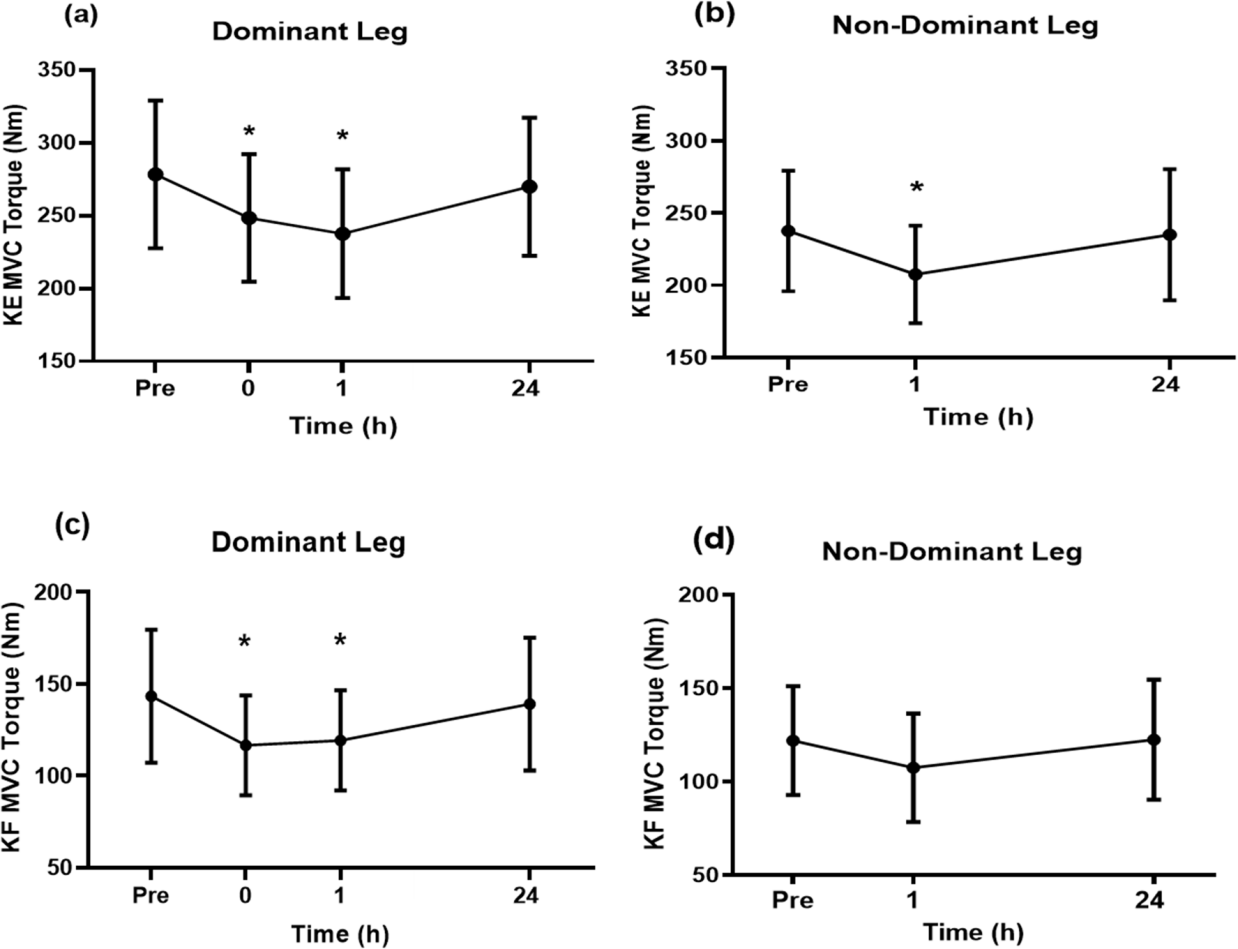
Changes (mean ± SD of 40 matches) in maximal voluntary isometric contraction (MVC) torque of the knee extensors (KE) of the dominant leg (**a**) prior to match (Pre), within 10 min post-match (0), and 1 h and 24 h post-match, and of the non-dominant leg (**b**) prior to match (Pre) and 1 h and 24 h post-match. Changes (mean ± SD) in MVC torque of the knee flexors (KF) of the dominant leg (**c**) prior to match (Pre), within 10 min post-match (0), and 1 h and 24 h post-match, and of the non-dominant leg (**d**) prior to match (Pre) and 1 h and 24 h post-match. * Significantly different from Pre

In average, knee extension MVC torque for the dominant leg was 278.4 ± 50.8 (185.0–351.3) Nm before the match, and decreased (*p* < 0.05) by 12.7 ± 2.9 (6.0–15.9)% immediately post-match and a further 4.0 ± 3.1 (0.9–10.9)% decrease at 1 h post-match, before recovering to baseline at 24 h post-match (Fig. 3a). As shown in Fig. 3b, knee extension MVC torque of the non-dominant leg was 237.6 ± 41.7 (155.5–299.3) Nm before the match, and decreased (*p* < 0.05) by 12.4 ± 4.1 (5.1–21.9)% at 1 h post-match (no measurement was taken immediately post-match), and recovered to baseline by 24 h post-match.

Knee flexion MVC torque for the dominant leg was 143.0 ± 36.2 Nm (88.3–201.5 Nm) before the match, and decreased (*p* < 0.05) by 18.0 ± 8.1 (10.3–38.6)% immediately post-match, and remained below baseline (*p* < 0.05) by 16.0 ± 7.4 (5.3–28.1)% at 1 h post-match, but recovered by 24 h post-match (Fig. 3c). Knee flexion MVC torque for the non-dominant leg was 122.0 ± 29.7 (93.1–152.4) Nm before the match, and no significant changes were observed post-match (Fig. 3d).

A significant interaction effect (*p* < 0.05) was observed for changes in MVC torque between knee extensors and flexors of the dominant leg, with the magnitude of decrease being greater for the flexors (17.9 ± 8.1%) than the extensors (10.6 ± 2.5%) immediately post-match. When comparing between the dominant and non-dominant legs, the magnitude of decrease in MVC torque was greater (*p* < 0.05) for the dominant than for non-dominant leg for both knee extensors (14.6 ± 3.2% vs 12.4 ± 4.1%) and flexors (16.0 ± 7.4% vs 12.5 ± 6.2%) at 1 h post-match.

#### Voluntary activation (VA)

**Fig. 4:**
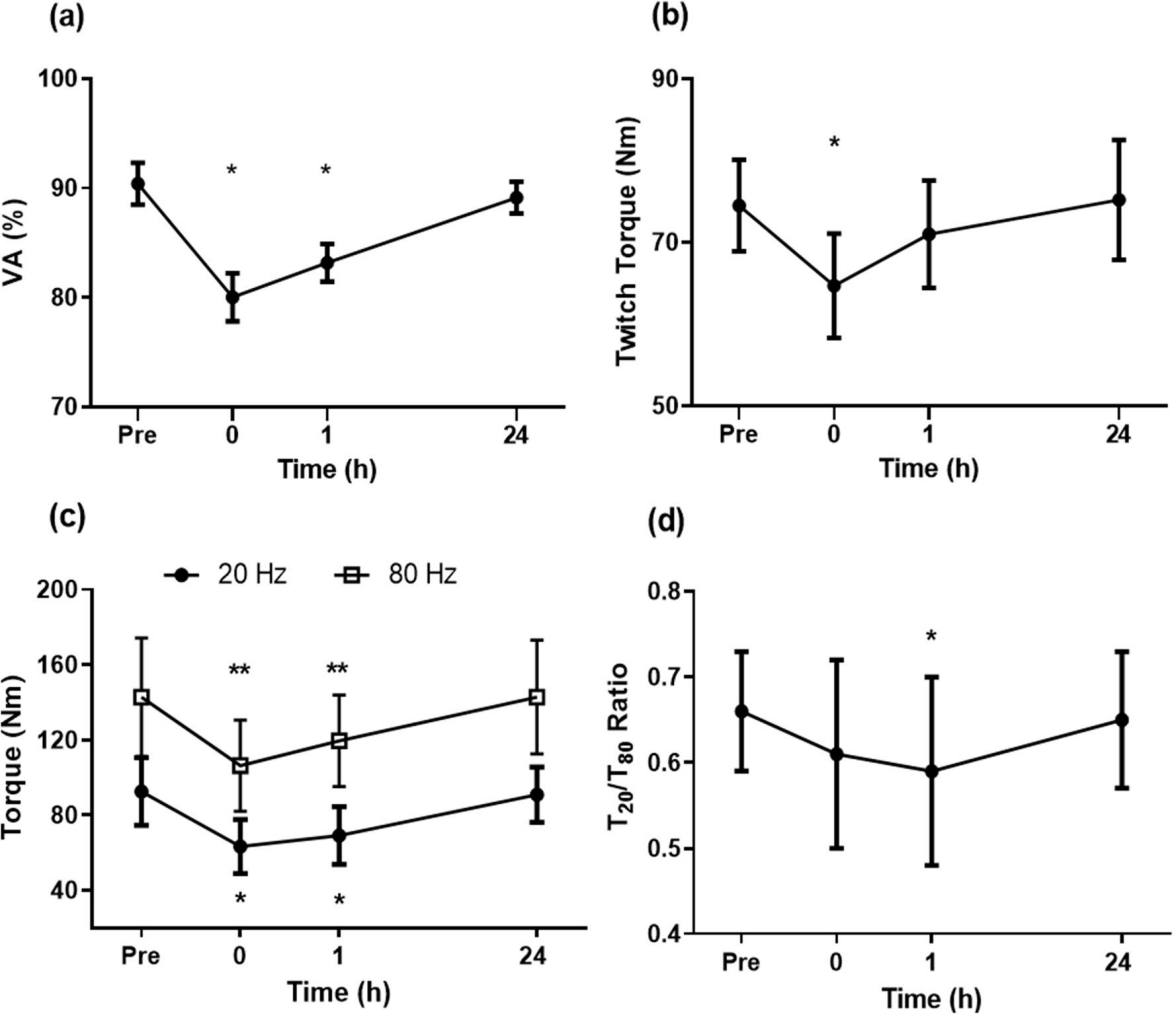
Changes (mean ± SD of 40 matches) in voluntary activation (VA) (**a**), torque doublet (*T*_doublet_) (**b**), torque induced by 20 Hz (*T*_20_) and 80 Hz (*T*_80_) stimulation (**c**) and *T*_20_/*T*_80_ ratio (**d**) of the dominant leg, prior to match (Pre), within 10 min post-match (0), and 1 h and 24 h post-match. * Significantly different from Pre

Knee extensor VA of the dominant leg was 90.4 ± 1.9 (87.3–94.2)% before the match, and decreased significantly (*p* < 0.05) by 11.4 ± 2.9% to 80.0 ± 2.2 (75.2–85.2)% at immediately post-match and by 8.0 ± 2.6% to 83.2 ± 1.7 (79.7–86.7)% at 1 h post-match, but returned to the baseline level by 24 h post-match (Fig. 4a).

#### Electrically evoked contractions (T_Doublet_)

*T*_Doublet_ and torque generated during *T*_20_ and *T*_80_ stimulations changed significantly over time. *T*_Doublet_ was 75.0 ± 5.6 (61.7–84.3) Nm at baseline and decreased (*p* < 0.05) by 13.1 ± 6.1% at immediately post-match, but returned to baseline by 1 h post-match (Fig. 4b). Torques induced by *T*_20_ and *T*_80_ stimulations were 92.5 ± 18.1 (57.2–122.1) Nm and 142.8 ± 31.6 (77.4–195.3) Nm, respectively, at baseline. *T*_20_ and *T*_80_ decreased (*p* < 0.05) by 31.1 ± 12.3 (11.4–45.6)% and 25.5 ± 7.9 (12.5–43.9)%, respectively, immediately post-match, and 24.3 ± 14.6 (9.6–41.9)% and 16.2 ± 8.1 (12.1–25.4)%, respectively, at 1 h post-match, but both recovered to baseline by 24 h post-match (Fig. 4c). The ratio between *T*_20_ and *T*_80_ (*T*_20_:*T*_80_) was 0.66 ± 0.07 (0.55–0.76) at baseline, and decreased significantly (*p* < 0.05) by 10.3 ± 13.3 (-3.4–30.6)% at 1 h post-match, and returned to baseline by 24 h post-match (Fig. 4d).

### Muscle soreness

**Fig. 5:**
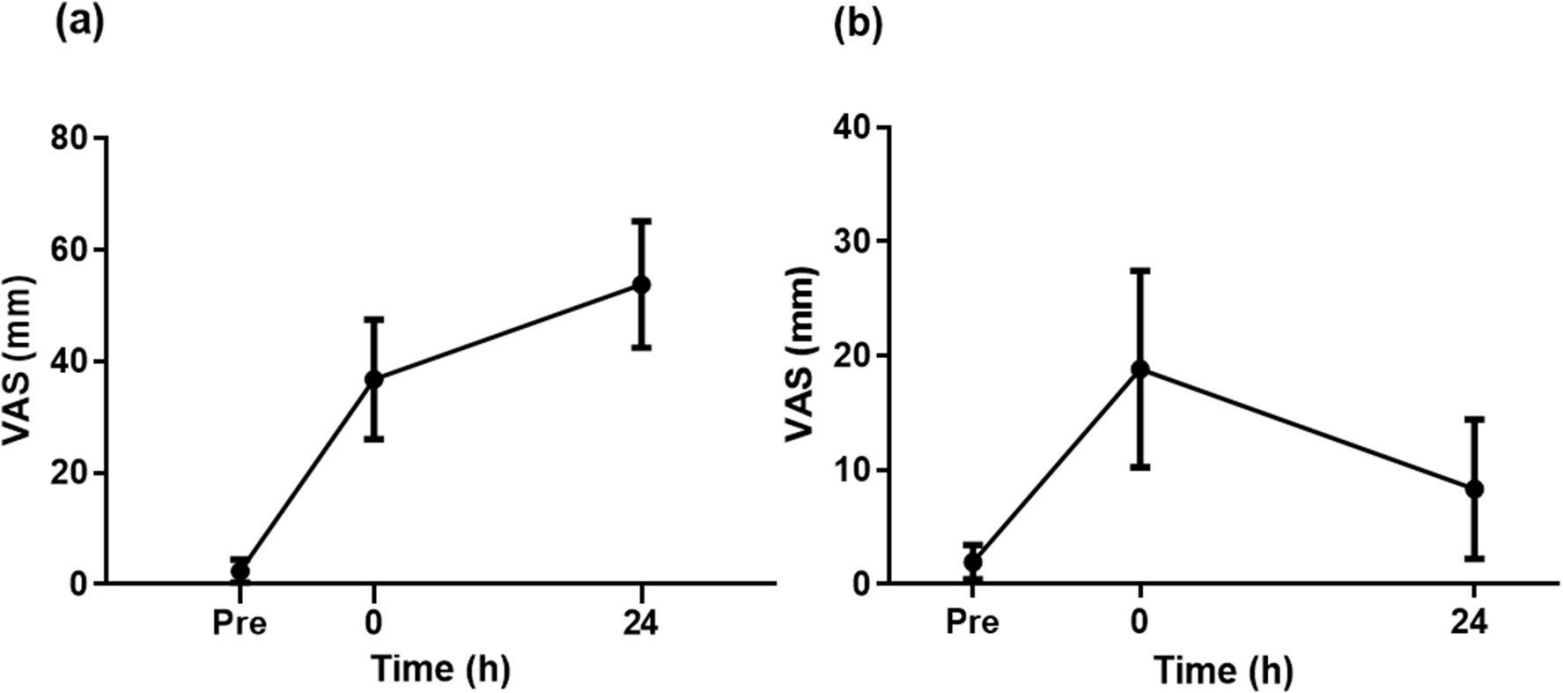
Changes (mean ± SD of 40 matches) in visual analogue scale for muscle pain for the dominant (**a**) and non-dominant leg (**b**) prior to match (Pre), within 10 min post-match (0), before (Pre) and 1 h and 24 h post-match. * Significantly different from Pre

VAS of the dominant leg was 2.4 ± 2.1 (0–7) mm at pre-match, and increased (*p* < 0.05) to 34.4 ± 11.2 (22–62) mm immediately post-match, and further increased (*p* < 0.05) to 51.5 ± 11.6 (34–80) mm at 24 h post-match (Fig. 5a). VAS for the non-dominant leg was 1.9 ± 1.5 (0–5) mm at pre-match and increased (*p* < 0.05) to 18.8 ± 8.6 (9–42) mm immediately post-match, but recovered to the pre-match value by 24 h post-match (Fig. 5b).

### Lunges

The total number of lunges performed in a match ranged 160–240 among matches per participant, with an average of 194 ± 18. Of the total number of lunges performed, 153 ± 12 (135–173) were deemed to be half lunges (i.e. the forward movement of the knee did not exceed the position of the toe) and 41 ± 15 (27–66) were deemed to be full lunges (the forward movement of the knee went beyond the toe).

**Fig. 6:**
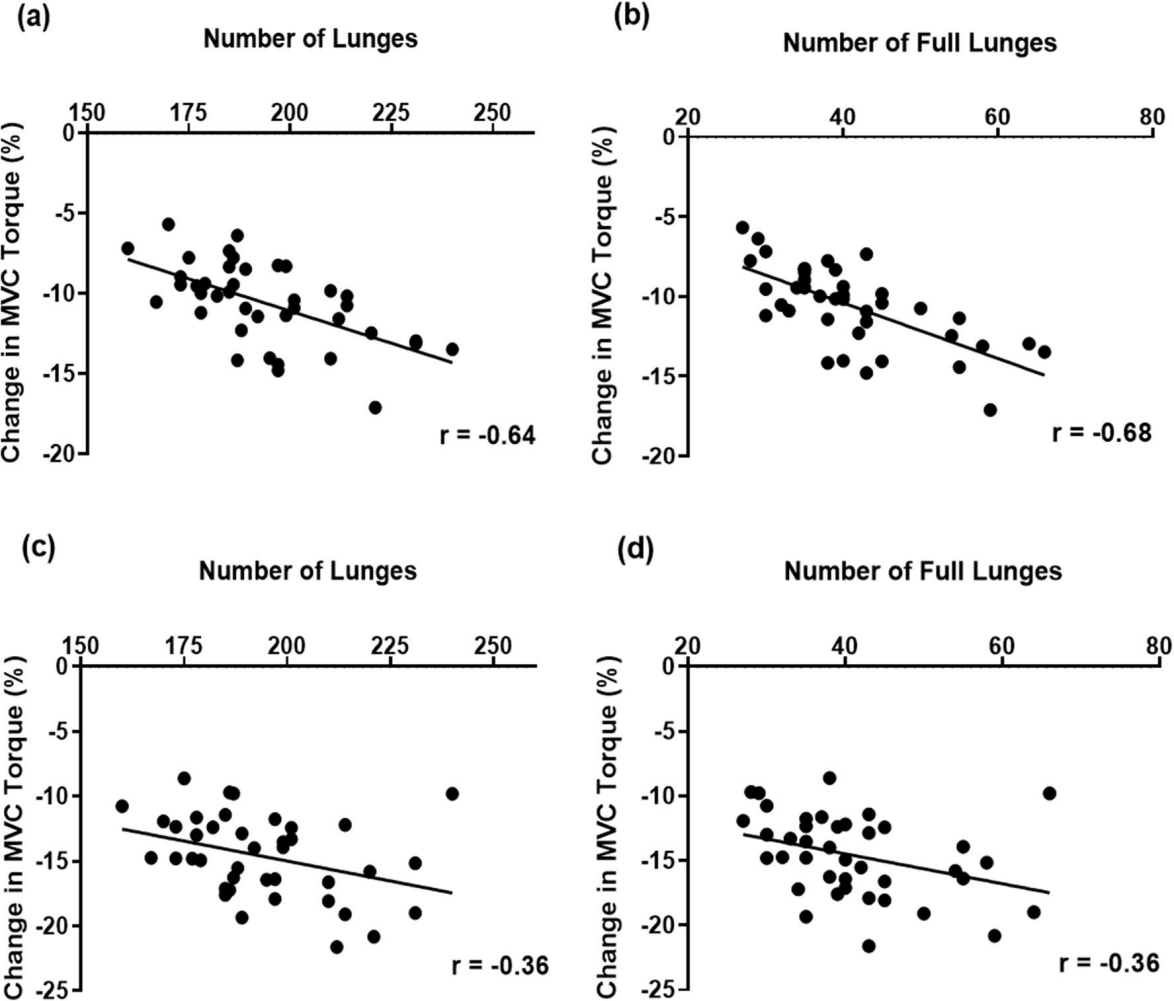
Correlation between the number of lunges and the magnitude of change in maximal voluntary isometric contraction (MVC) torque of the knee extensors of the dominant or non-dominant leg (%) from pre- to immediately post-match (dominant leg: **a**, non-dominant leg: **c**), and correlation between the number of full lunges and the magnitude of change in MVC torque of the knee extensors of the dominant or non-dominant leg (%) from pre- to immediately post-match (**b**, **d**)

A significant correlation (*r* = − 0.64, *p* < 0.001) was observed between the total number of lunges and the magnitude of change in knee extensor MVC torque of the dominant leg from pre- to immediately post-match (Fig. 6a). A significant correlation (*r* = − 0.68, *p* < 0.01) was also found between the number of full lunges and the magnitude of change in knee extensor MVC torque from pre- to immediately post-match (Fig. 6b). For the non-dominant leg, a lower but significant correlation was observed between the number of total lunges (*r* = − 0.36, *p* < 0.05) or full lunges (*r* = − 0.39, *p* < 0.05) and the magnitude of change in knee extensor MVC torque from pre- to 1 h post-match (Fig. 6c and d).

## Discussion

The present study tested the hypotheses that the knee extensor and flexor muscle strength would be decreased for at least 1 day after a simulated badminton match, and that the magnitude of changes in the muscle strength and neuromuscular parameters would be associated with the number of lunges performed in the match, in well-trained badminton players who were in their competition phase and had played multiple matches prior to entering the study. In contrast to the hypothesis, the results showed that knee extensor and flexor MVC torques of the dominant leg decreased immediately (− 11% and − 18%, respectively) and 1 h post-match (− 14% and − 16%, respectively), but returned to baseline by 24 h post-match. The decrease was accompanied by significant reductions in voluntary knee extensor activation, and the torques evoked by electrical stimulations (VA [11.4%], *T*_doublet_ [13.1%], *T*_20_ [31.1%] and *T*_80_ [25.5%]). However, at 24 h later, these measures had returned to pre-match levels. These changes were less than those reported after other racket sports such as tennis (Girard et al. 2006) and squash (Girard et al. 2010).

In the present study, the average *V*O_2max_ was 57 ml min^−1^ kg^−1^, which was similar to that of internationally ranked singles badminton players reported in a previous study (Faude et al. 2007). Previous studies (Faude et al. 2007; Heller 2010; Ooi et al. 2009) reported that the average heart rate was ~ 90% of the maximum heart rate during a match, which was similar in the current study. CR-10 was 7.9 at post-match, indicating that the 1-h simulated badminton match was ‘hard’ to ‘very hard’. In the present study, the blood lactate concentrations increased to 7.2 ± 1.3 mM l^−1^ on average post-match, which were similar to those reported by Faude et al. (2007). Thus, the physiological stress of the simulated matches appears to be similar to that imposed by actual matches. The results obtained using the present experimental design, therefore, appear to be valid indicators of the changes occurring in real competitions.

Peak knee extensor and flexor MVC torques of the dominant leg decreased at immediately post-match by 11% and 15%, respectively, and they did not recover at 1 h post-match (Fig. 3). This was accompanied by decreases in voluntary activation (immediately post-match: − 12%, 1 h post-match: − 8%) and knee extensor peak doublet torque (− 13%, − 4%) as shown in Fig. 4, indicating that both “central” and “peripheral” fatigue were induced. Peak knee extensor torque in the non-dominant leg was also decreased (− 12%) at 1 h post-match, although the magnitude of decrease was significantly smaller than that in the dominant leg (Fig. 3). This was likely related to the more execution of lunges by the dominant leg, but other movements (e.g. acceleration, deceleration, change of direction) were likely to have been performed by both legs similarly. It should be noted that MVC torque and other muscle function variables returned to pre-match levels by 24 h post-match. These suggest that muscle damage induced by the badminton matches was minimal, since a prolonged loss of muscle function lasting more than 1 day is considered to indicate muscle damage (Girard and Millet 2009). Hence, it seems likely that similar levels of play can be performed with little effects on performances in a match at 24 h later.

It should be noted that knee extensor MVC torque decreased further from immediately post- to 1 h post-match, and this was accompanied by greater decreases in *T*_20_ than *T*_80_, suggesting low-frequency fatigue, an indicative of excitation–contraction coupling failure (Girard and Millet 2009; Girard et al. 2010). Interestingly, knee extensor MVC torque did not recover after 1 h, and thus, based on the reduction in muscular activity and lack of drive reduction from the electrical evoke stimulations, suggesting the presence of central fatigue. This is supported by the fact that voluntary activation capacity was still significantly reduced at 1 h post-match, possibly reflecting impairment to central drive. This reduction might result from the effect of pain on III/IV afferents, resulting in inhibition at the spinal level and reduction in drive to the muscle (Gandevia 2001; Girard et al. 2010). However, it should be noted that MVC torque returned to the baseline at 24 h post-match when muscle soreness was higher. It may be that a more general ‘central fatigue’ resulted from the III/IV afferent effects supraspinally, as suggested by Girard and colleagues (Girard et al. 2010). If this was the case, we might expect fatigue of non-exercised muscles. However, this was not found such that grip strength did not change after matches. Therefore, it is most likely that the loss of maximum force in the leg muscles resulted from alterations in local circuits. In fact, *T*_20_ and *T*_80_ decreased immediately and 1 h post-match, but recovered to baseline by 24 h post-match (Fig. 4c).

It should also be noted that the magnitude of MVC torque decrease was greater for the dominant than non-dominant leg, which is suggestive that the performance of lunges during the match affected MVC torque. The total number of lunges and full lunges was significantly correlated with the magnitude of decrease in knee extensor torque of the dominant leg at immediately post-match (Fig. 6). This suggests that the execution of lunges, especially full lunges, was a cause of fatigue leading to the decreases in the MVC torque. Although no significant correlation was observed between the number of lunges and the magnitude of decrease in knee flexor MVC torque in the dominant leg, a reduction of MVC torque of the knee flexors was evident (Fig. 3). Lees (2003) stated that the deceleration during the initial loading phase of the lunge action and acceleration during the recovery of the lunge could possibly contribute to overall muscle function loss. This might explain the decrease in the knee flexor MVC torque. Future studies should examine whether resistance training of the lower extremities (e.g. knee extensors and flexors) helps to prevent DOMS and loss of muscle function, or whether it is more effective than simply playing additional badminton matches. It is also beneficial to establish a simple method that can be used close to a court to assess muscle function in relation to fatigue and muscle damage as an alternative to the use of an isokinetic dynamometer in a laboratory setup.

Muscle soreness increased in both legs immediately post-match with a further increase observed only in the dominant leg at 24 h post-match (Fig. 5), but the level of pain was moderate and was unlikely to have been particularly irritating to the players. It was notable that the participants still experienced moderate muscle soreness at 24 h post-match. Since they had been playing matches regularly, and they played eight matches over 4 weeks in the present study, muscle soreness could have been less due to the repeated bout effect (Hyldahl et al. 2017). It has been reported that muscle soreness is more related to inflammation of connective tissues surrounding the muscle fibres and fascicles (i.e. endomysium, perimysium) than muscle fibre damage (Nosaka et al. 2001; Paulsen et al. 2010). It is possible that the connective tissues were damaged and inflamed during badminton matches, evoking DOMS. Importantly, the pain did not cause a loss of muscle force production as shown the recovery of MVC torque at 24 h post-match. Moreno-Perez et al. (2020) investigated the effects of consecutive badminton competition matches in a single day on muscle damage in elite junior badminton players and reported that hip range of motion was reduced and creatine kinase activity remained elevated at 24 h post-match despite hip muscular strength increasing after the consecutive competition matches. The researchers postulated that muscle damage did not affect the muscle performance negatively, but that some form of physical conditioning was still important to minimise it. Since a decrease in MVC torque remained for at least 1 h post-match in the present study (Fig. 3), effective recovery strategies appear to be necessary to ensure optimal recovery of the lower limb muscles if another match or a training session is scheduled to be played on the same day.

There were limitations in the current study. First, the players were well trained and considered to be high level, but were not national or international levels, and the nature of the game (1-h simulated match) may not represent a competitive environment such as in a tournament. Thus, the results of the present study cannot be generalised to all badminton singles matches and players. Second, the interval between exercise cessation and commencement of the neuromuscular assessments (within 10 min) may have allowed some recovery. Hence, the “immediately post-match” results may not fully represent the exact condition of immediately post-exercise. This delay was due to the distance between the gym to play badminton and the laboratory where the isokinetic dynamometer was located. However, in the present study, a prolonged loss of muscle function, which is an indicator of muscle damage, was also important, which was captured by the time points at 1 h and 24 h post-match. The rate of force development and muscle activity by EMG measures during MVC were not included in the present study. These should be investigated in future studies. It is also necessary to establish a simple method to assess changes in muscle function in relation to fatigue and muscle damage, since an isokinetic dynamometer is not available in a practical situation. Thus, measurements that could be performed easily but are reliable and valid, should be established. Third, due to the methodological limitations, most of the measurements were taken from one of the two players in a match. Although the same pairs played against twice, the match characteristics were likely to be different between them. Thus, it would have been better to take the measurements from the two players who played in the same match. Fourth, the wearing of the device might restrict certain movements during activity; hence, it may be best to allow more time for familiarisation of the device to allow the best possible result to be achieved during the actual activity measurement. Lastly, in the video analyses for the number of lunges, the movement velocities before lunges, and movements after lunges were not considered. Thus, the “intensity” of the lunges which might be associated with the intensity of “eccentric contractions” was not known. It is interesting to analyse lunges and lunging ability in badminton in future studies.

In conclusion, muscle damage induced by singles matches was minimal in this well-trained cohort, and post-match functional decrements were fully recovered by 24 h post-match. Therefore, it is unlikely that residual fatigue or muscle damage would impact on match performance on the following day; one caveat is that minor muscle soreness was still felt at 24 h post-match, which may be a concern for some. The number of lunges performed in a match was associated with the magnitude of neuromuscular fatigue, suggesting the possibility that fatigue could be substantive if a player were required to perform a very large number of lunges. This fatigue appeared to have both central and peripheral origins. However, specific mitigation practices (additional to formal physical training methodologies) may not be needed unless repeated matches or training sessions are performed on the same day; in these cases, interventions that minimise central aspects of fatigue may be of potential benefit.

## Data Availability

Data available on request from the authors.

## Abbreviations

ANOVA: Analysis of variance
CR-10: Borg’s category ratio-scale 10
DOMS: Delayed onset muscle damage
EMG: Electromyography
HR: Heart rate
MVC: Maximal voluntary contraction
RPE: Rating of perceived exertion
*T*_Doublet_: Torque by doublet electrical stimulation
*T*_20_: Torque by 20 Hz electrical stimulation
*T*_80_: Torque by 80 Hz electrical stimulation
VA: Voluntary activation
VAS: Visual analogue scale
*V*O_2_: Volume of oxygen consumption
